# Supplementary material for: Anti-Kasha triplet energy transfer and excitation wavelength dependent persistent luminescence from host-guest doping systems
Source: Nat Commun. 2023 Dec 7;14:8098. doi: 10.1038/s41467-023-43687-0 (PMC10703808; doi:10.1038/s41467-023-43687-0)
Supplement: Supplementary file 1 — Supporting Information [file 41467_2023_43687_MOESM1_ESM.pdf]

# Supplementary Information

# Anti-Kasha Triplet Energy Transfer and Excitation Wavelength Dependent Persistent Luminescence from Host-Guest Doping Systems

Weiwei Xie[1†], Wenbin Huang[2†], Jietai Li[1], Zikai He[2]*, Guangxi Huang[1]*, Bing Shi Li[1]* and Ben Zhong Tang[3]*

[1]Key Laboratory of New Lithium-Ion Battery and Mesoporous Material, College of Chemistry and Environmental Engineering, Shenzhen University, 1066 Xueyuan Avenue, Nanshan District, Shenzhen, Guangdong 518055, China.

[2]School of Science, Harbin Institute of Technology, Shenzhen, HIT Campus of University Town, Shenzhen, 518055, China.

[3]School of Science and Engineering, Shenzhen Institute of Aggregate Science and Technology, The Chinese University of Hong Kong, Shenzhen, Guangdong 518172, China.

*Corresponding author. E-mail: phbingsl@szu.edu.cn; huanggx@iccas.ac.cn; hezikai@hit.edu.cn; tangbenz@cuhk.edu.cn.

†These authors contributed equally to this work.

**This PDF file includes:**

Supplementary Figure 1-26

Supplementary Table 1-3

Supplementary References

## Supplementary Figures and Tables

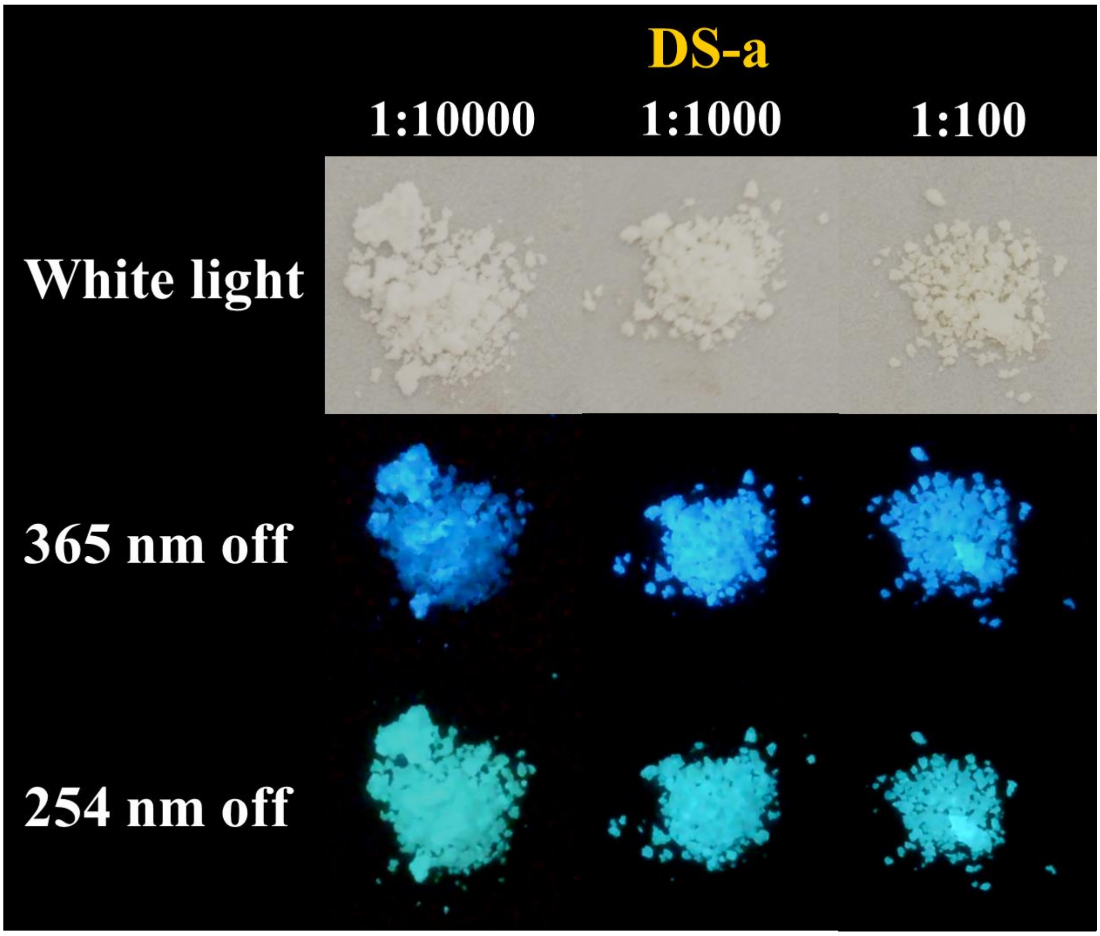


**Supplementary Figure 1** Images of **DS-a 1:10000**, **DS-a 1:1000** and **DS-a 1:100** before and after 365/254 nm UV irradiation under ambient conditions.

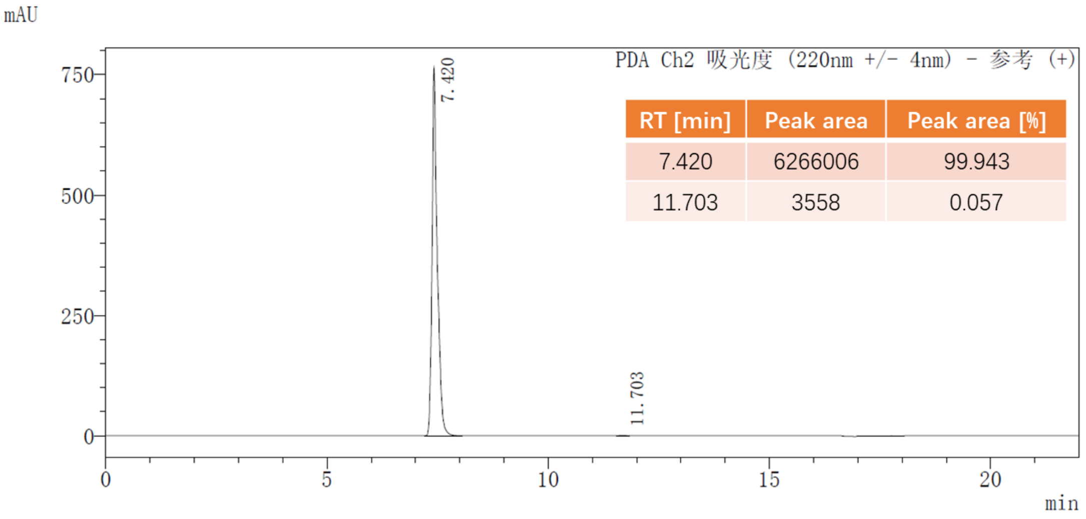


| Column | InfinityLabPorroshell 120EC-C18 0.46cm I.D.x10cm L,2.7μm |
|---|---|
| Injection | 2μl |
| Mobile phase | B: ACN; A: H2O(10mMNH4FA) (v/v) |
| Gradient | 0.00min. 20%B, 0.50min. 20%B, 10.00min. 90%B, 15.00min. 90%B, 16.00min. 20%B, 22.00min. 20%B (v/v) |
| Flow rate | 0.8 ml/min |
| Wavelength | UV220nm |
| Temperature | 30℃ |
| Sample solution | 1.0 mg/ml in CAN |
| HPLC equipment | Shimadzu UFLC30A QA&QC-HPLC-21 |

**Supplementary Figure 2** HPLC curve of **host** in acetonitrile/$H_2O$ solution and the measuring parameters.

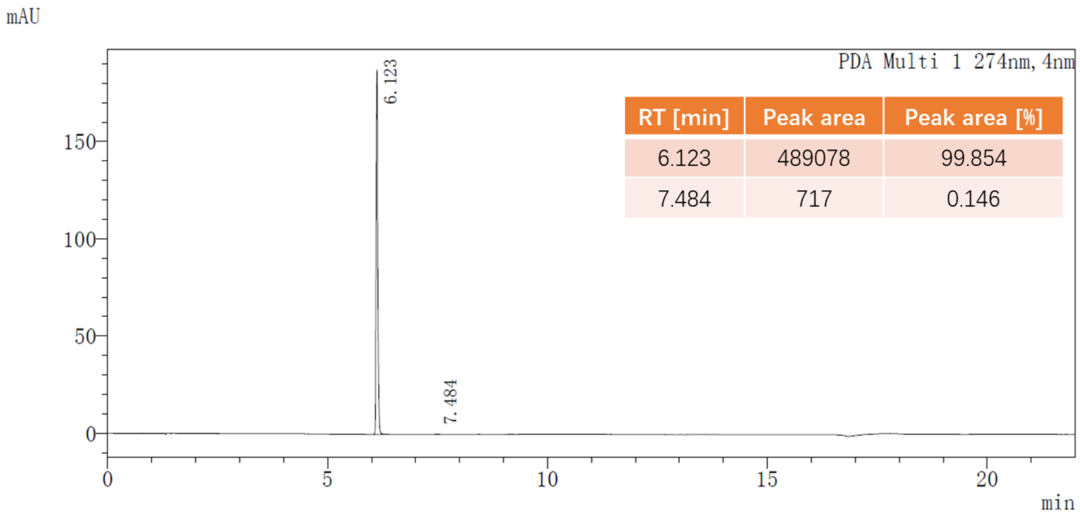


| Column | InfinityLabPorroshell 120EC-C18 0.46cm I.D.x10cm L,2.7µm |
|---|---|
| Injection | 0.5µl |
| Mobile phase | B： ACN； A： H2O(10mMNH4FA) (v/v) |
| Gradient | 0.00min. 10%B， 0.50min. 10%B， 10.00min. 90%B， 15.00min. 90%B， 16.00min. 10%B， 22.00min. 10%B (v/v) |
| Flow rate | 0.8 ml/min |
| Wavelength | UV274nm |
| Temperature | 30℃ |
| Sample solution | 0.7 mg/ml in ACN90DMSO10 |
| HPLC equipment | Shimadzu UFLC30A QA&QC-HPLC-21 |

**Supplementary Figure 3** HPLC curve of **guest** in acetonitrile/$H_2O$ solution and the measuring parameters.

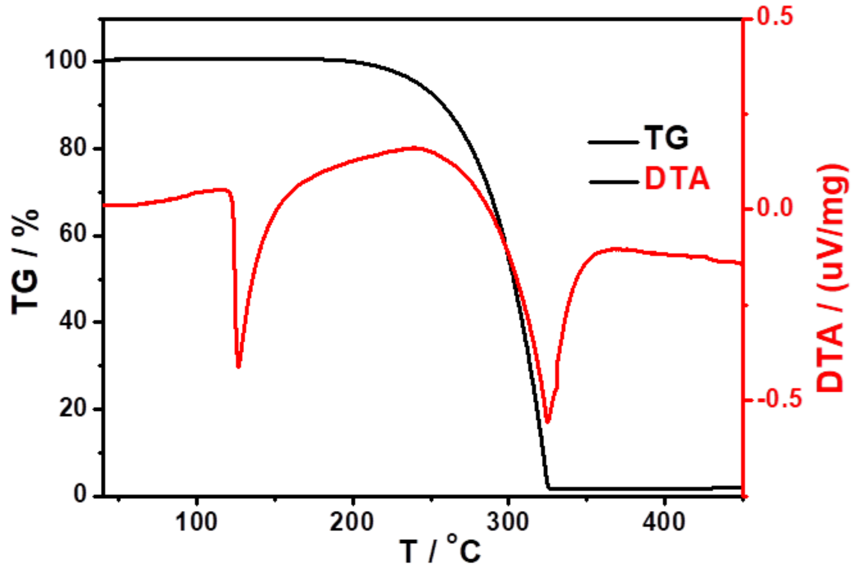


**Supplementary Figure 4** Thermogravimetric and DTA curves of **host**.

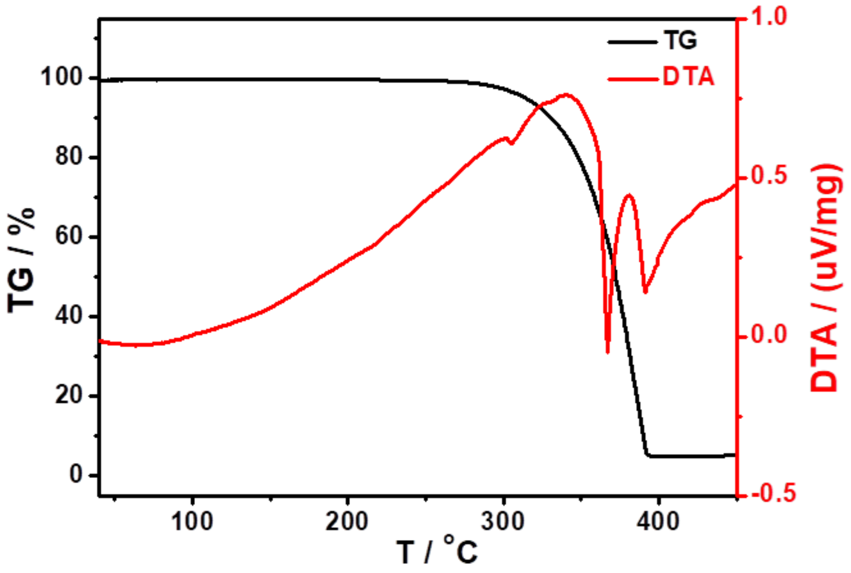


**Supplementary Figure 5** Thermogravimetric and DTA curves of **guest**.

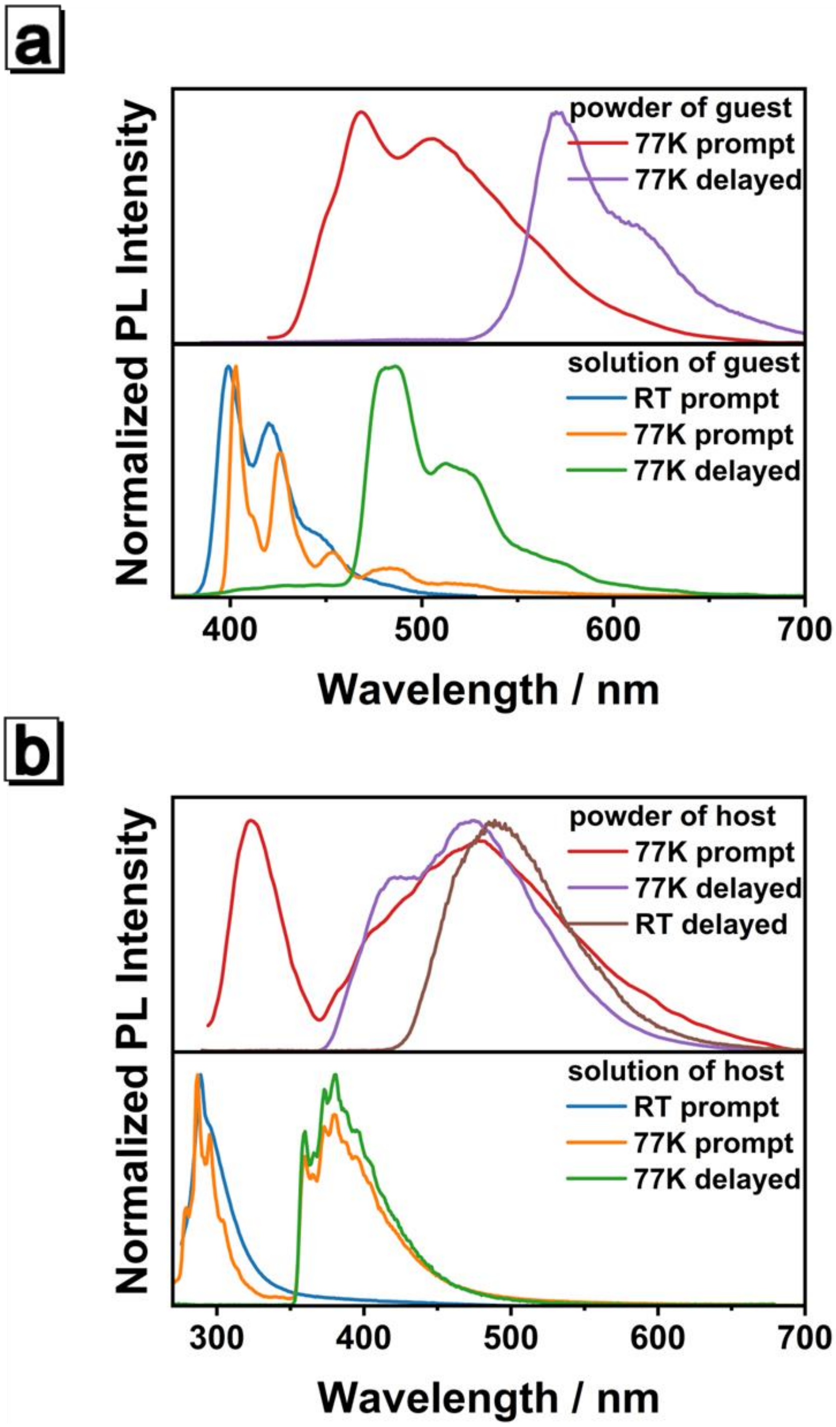


**Supplementary Figure 6** Normalized steady-state spectra and delayed PL spectra of (a) **guest** and (b) **host** in 2-MeTHF solution (10 μM) and solid state at 77K and 298K.

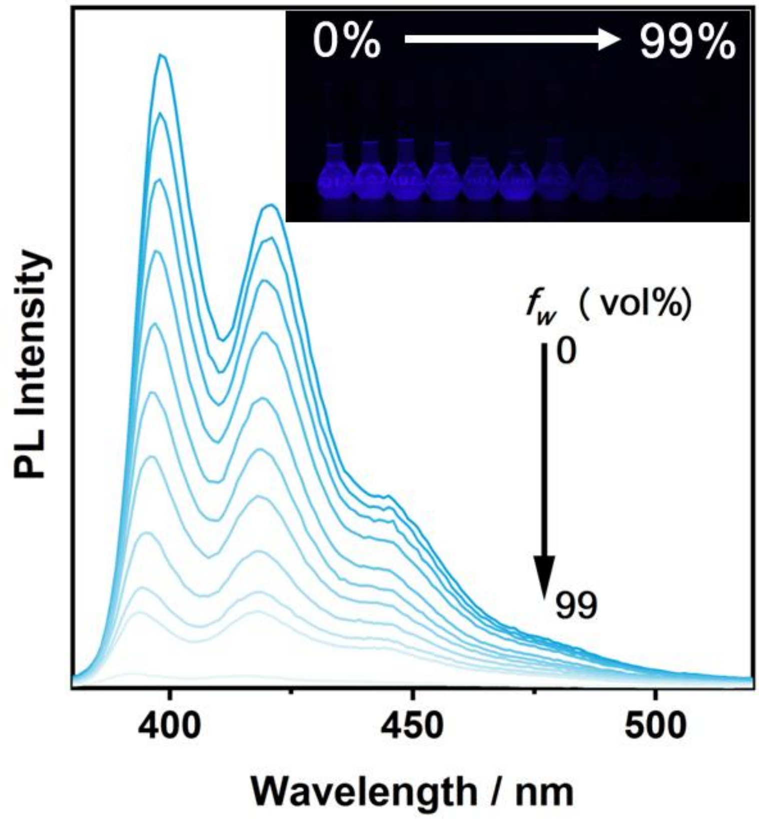


**Supplementary Figure 7** PL spectra of **guest** in n-hexane/THF mixtures with different n-hexane fractions $f_h$. Concentration: 10 μM.

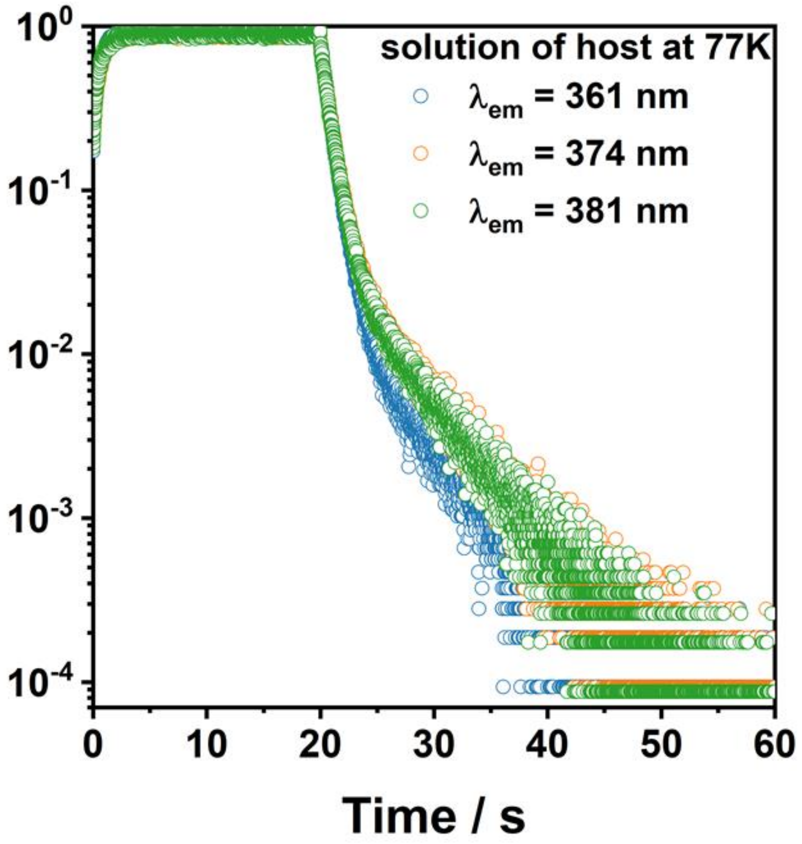


**Supplementary Figure 8** Decay curves of the delayed emission in the 2-MeTHF solution (10 μM) of **host** at 77 K.

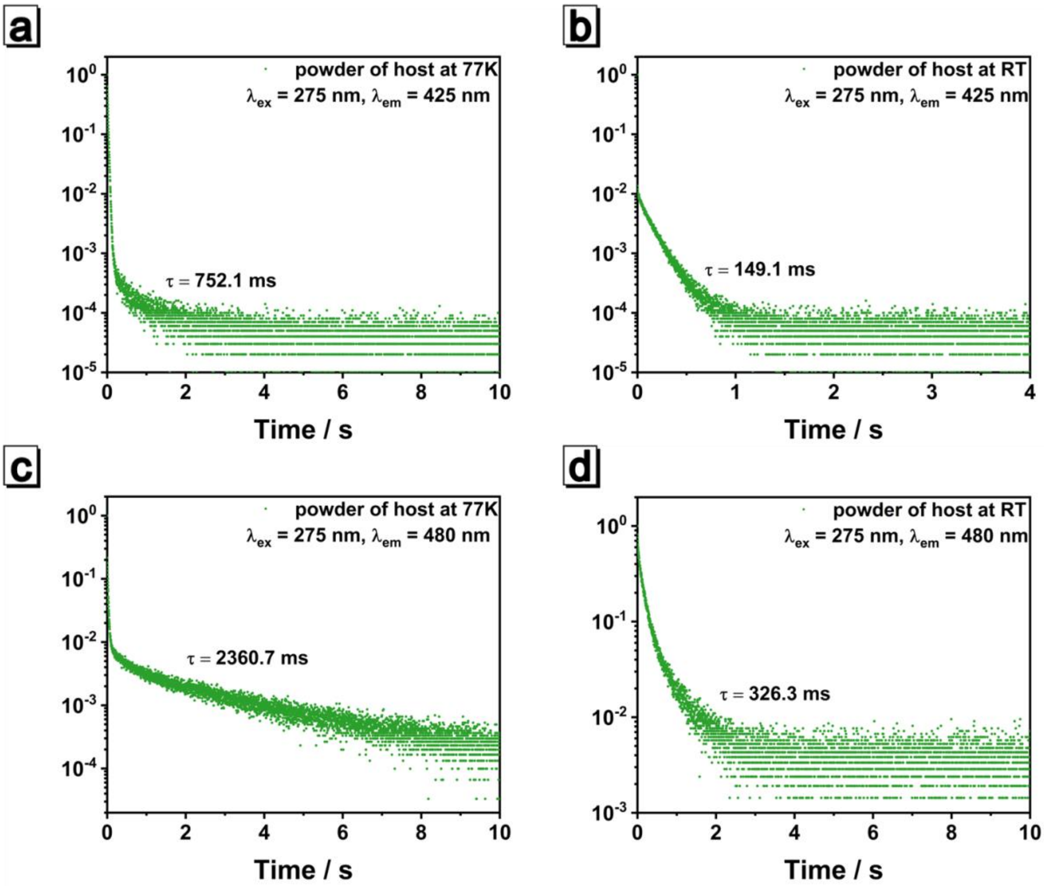


**Supplementary Figure 9** Decay curves at 425 nm delayed emissions of **host** powder at (a) and (c) 77 K. Decay curves at 480 nm delayed emissions of **host** powder at (b) RT and (d) 77 K..

Supplementary Figure 10 caption follows the figure.

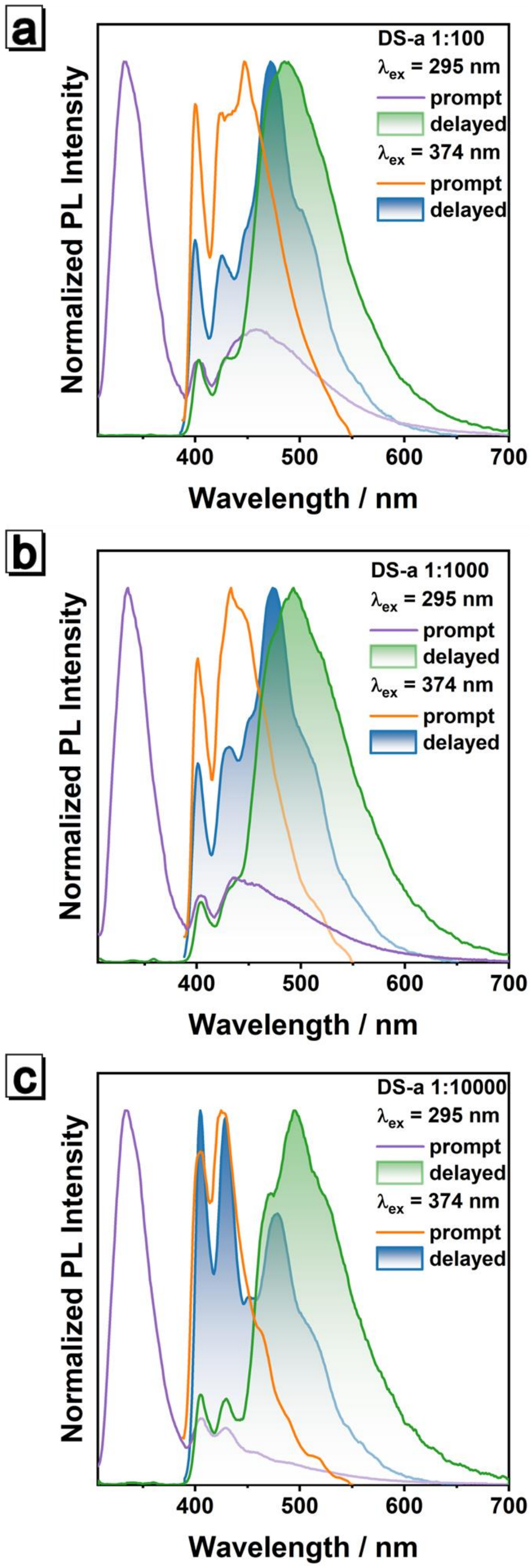


**Supplementary Figure 10** Normalized steady-state and delayed PL spectra of (a) **DS-a 1:100**, (b) **DS-a 1:1000** and (c) **DS-a 1:10000** under 295 nm or 374 nm excitation.

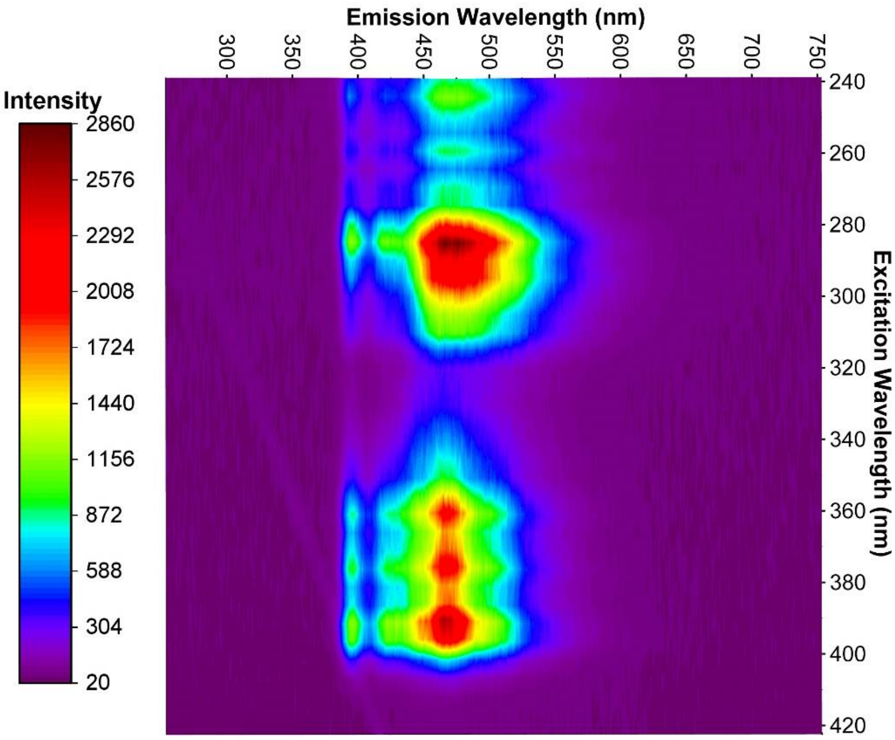


**Supplementary Figure 11** Excitation-afterglow mapping of **DS-a 1:1000** under ambient condition.

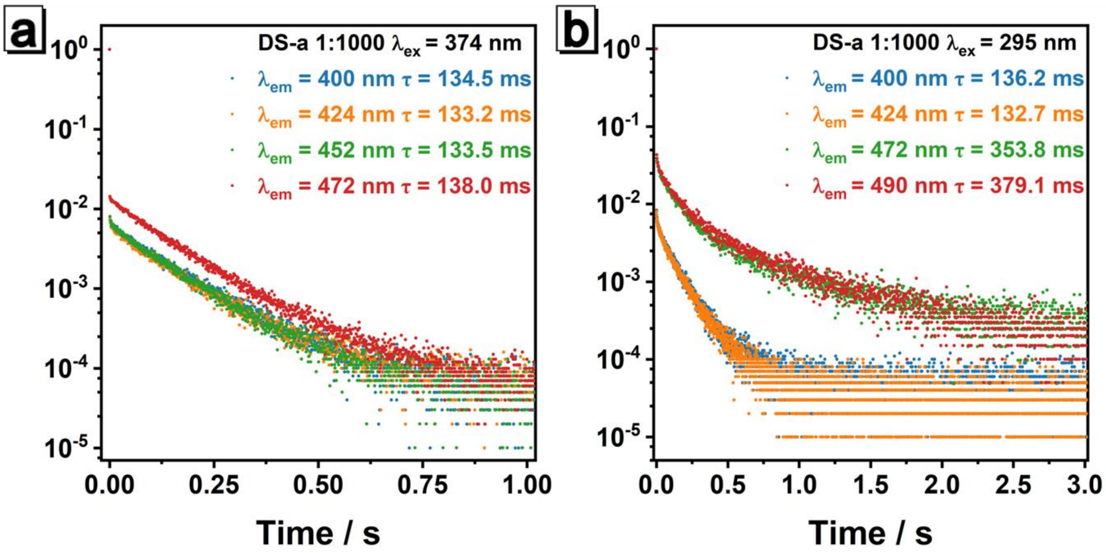


**Supplementary Figure 12** Decay curves of delayed emissions of **DS-a 1:1000** after (a) 374 nm and (b) 295 nm excitation.

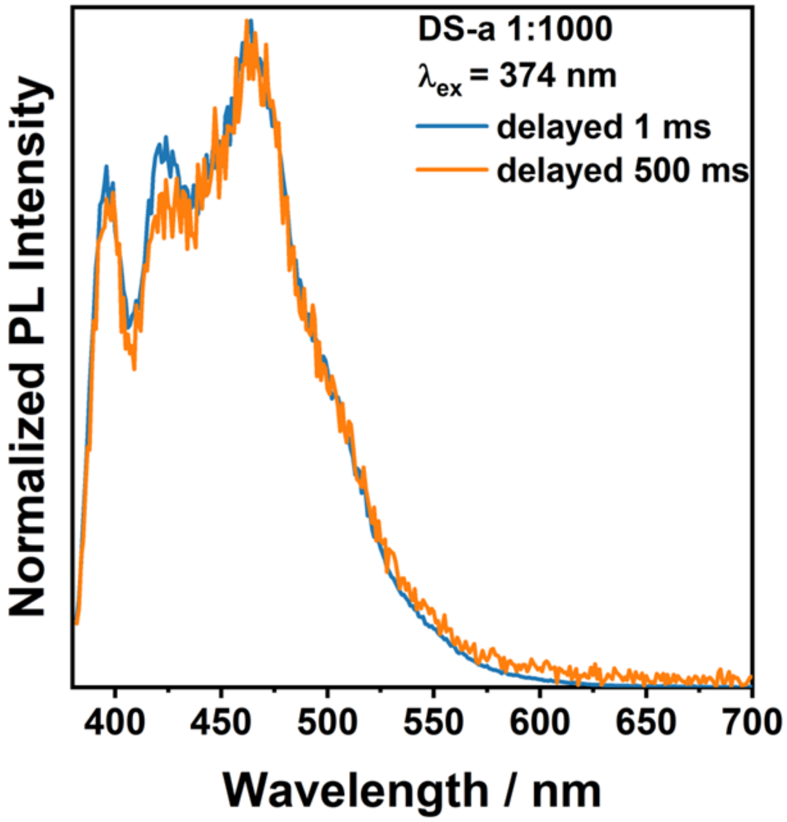


**Supplementary Figure 13** Normalized delayed PL spectra of **DS-a 1:1000** with different delayed times after 374 nm excitation.

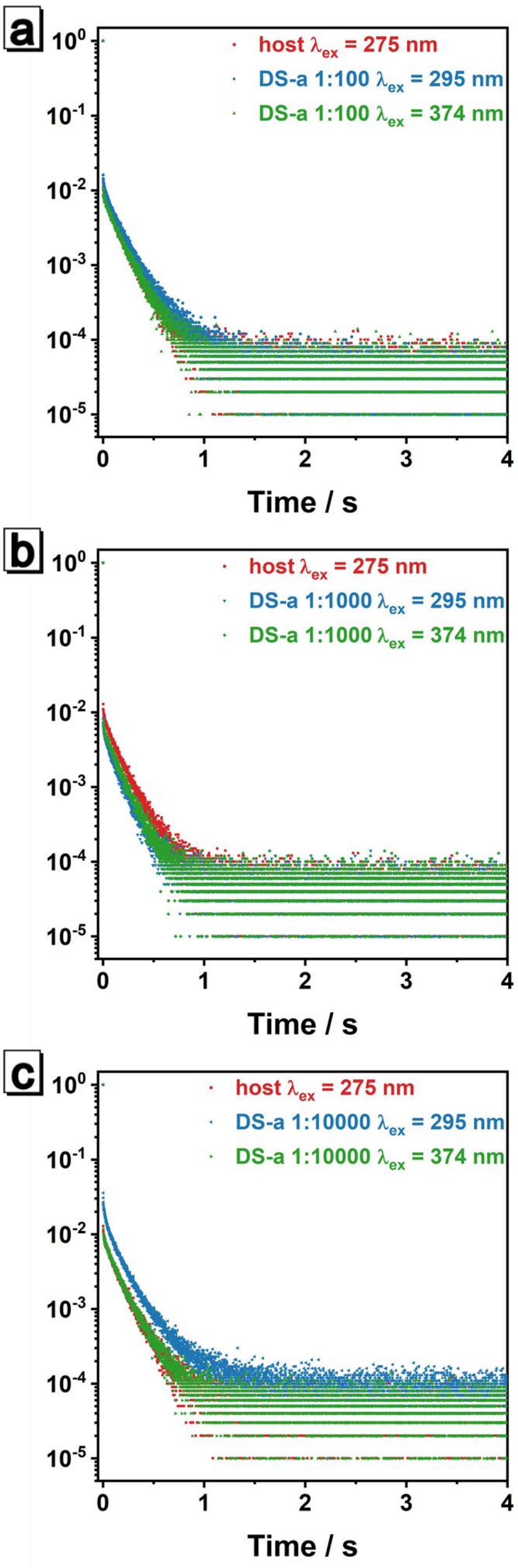


**Supplementary Figure 14** Decay curves of the emission at 400 nm of **host** powder under 275 nm excitation and (a) **DS-a 1:100**, (b) **DS-a 1:1000** and (c) **DS-a 1:10000** under 295 nm or 374 nm excitation.

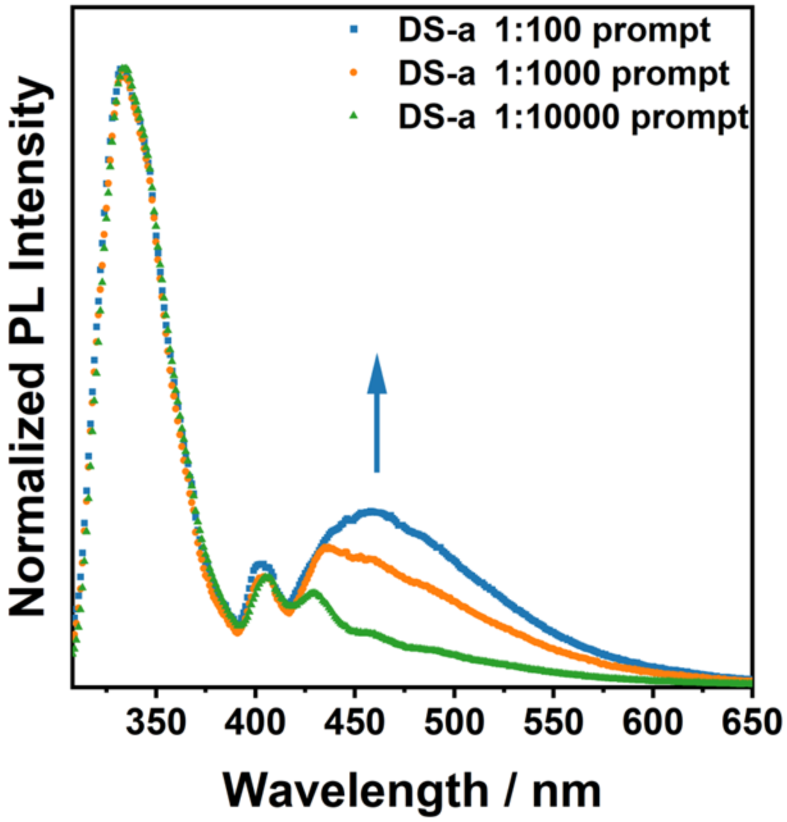


**Supplementary Figure 15** Normalized steady-state PL spectra of **DS-a** with different doping ratios.

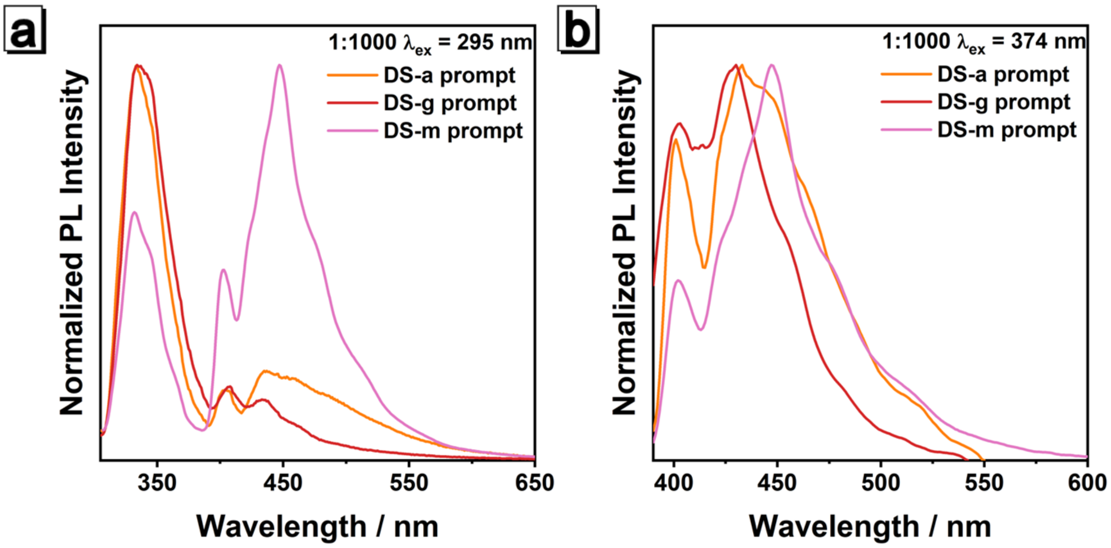


**Supplementary Figure 16** Normalized steady-state spectra of **DS-a**, **DS-m** and **DS-g** with a doping ratio of 1:1000 under (a) 295 nm and (b) 374 nm excitation.

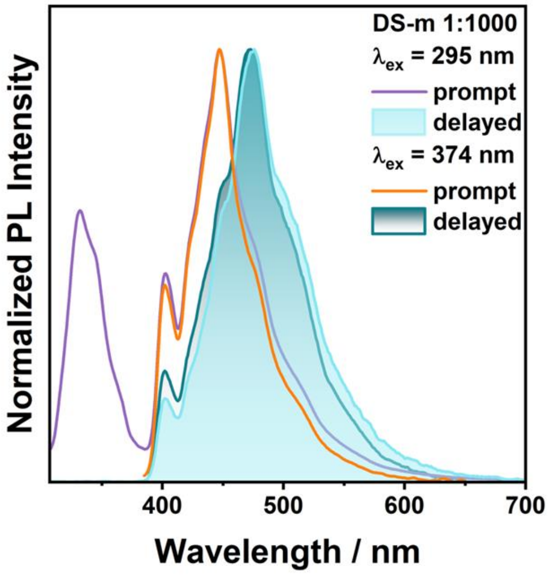


**Supplementary Figure 17** Normalized steady-state spectra and delayed PL spectra of **DS-m 1:1000** under 295 nm or 374 nm excitation.

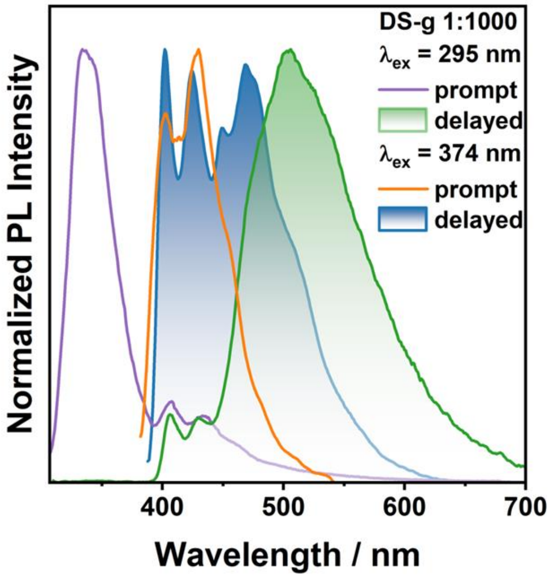


**Supplementary Figure 18** Normalized steady-state spectra and delayed PL spectra of **DS-g 1:1000** under 295 nm or 374 nm excitation.

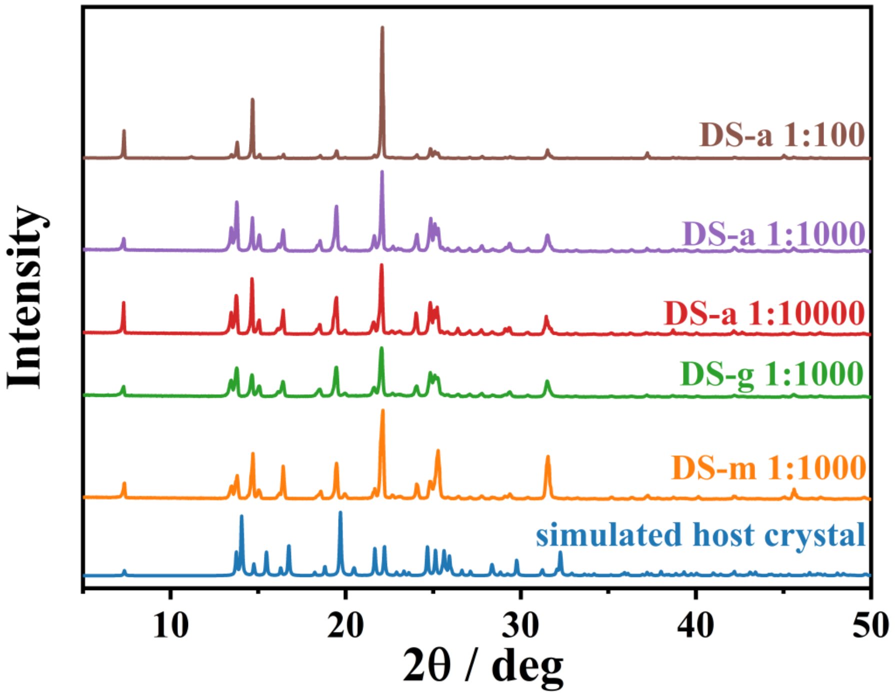


**Supplementary Figure 19** PXRD patterns of different doping samples and single crystal of **host**. Single crystal of **host** was prepared by slow evaporation of its dichloromethane/hexane (v/v = 1/1) solution at room temperature. The simulated PXRD pattern of **host** crystal derives from the attachment file "single crystal data of **host**". The same single crystal of **host** (CCDC number: 1905615) had ever been reported by Chi et al.[1]

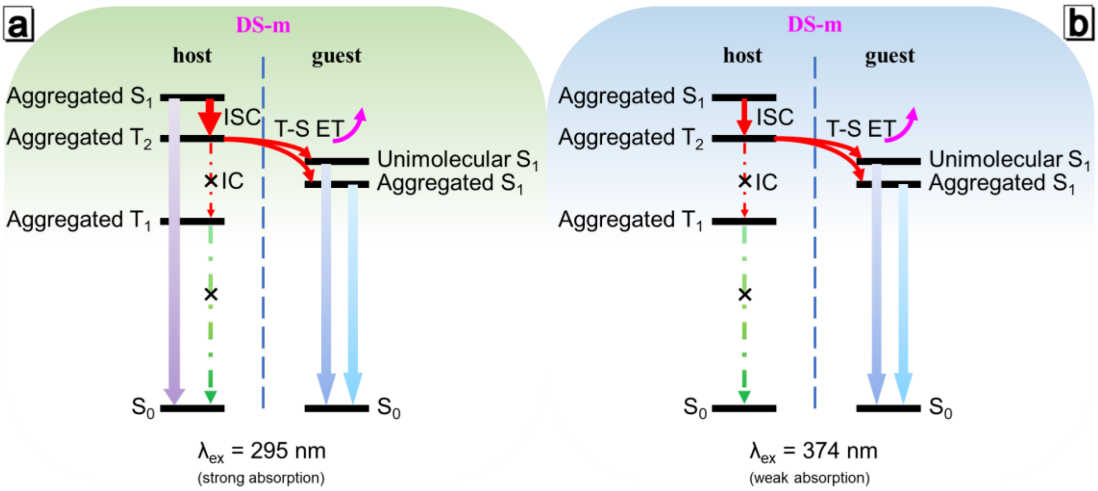


**Supplementary Figure 20** Proposed mechanism of photophysical process of **DS-m** under (a) 295 nm and (b) 374 nm excitation.

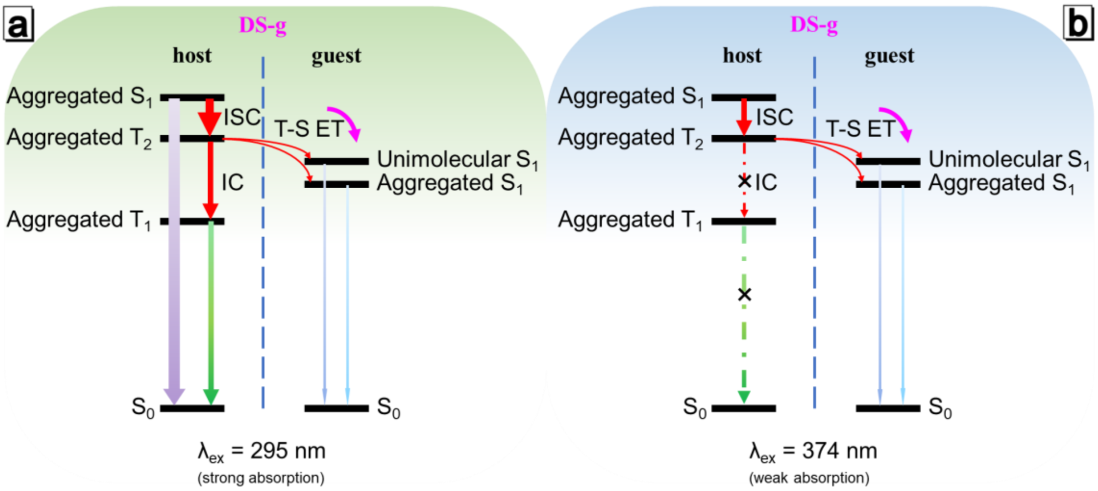


**Supplementary Figure 21** Proposed mechanism of photophysical process of **DS-g** under (a) 295 nm and (b) 374 nm excitation.

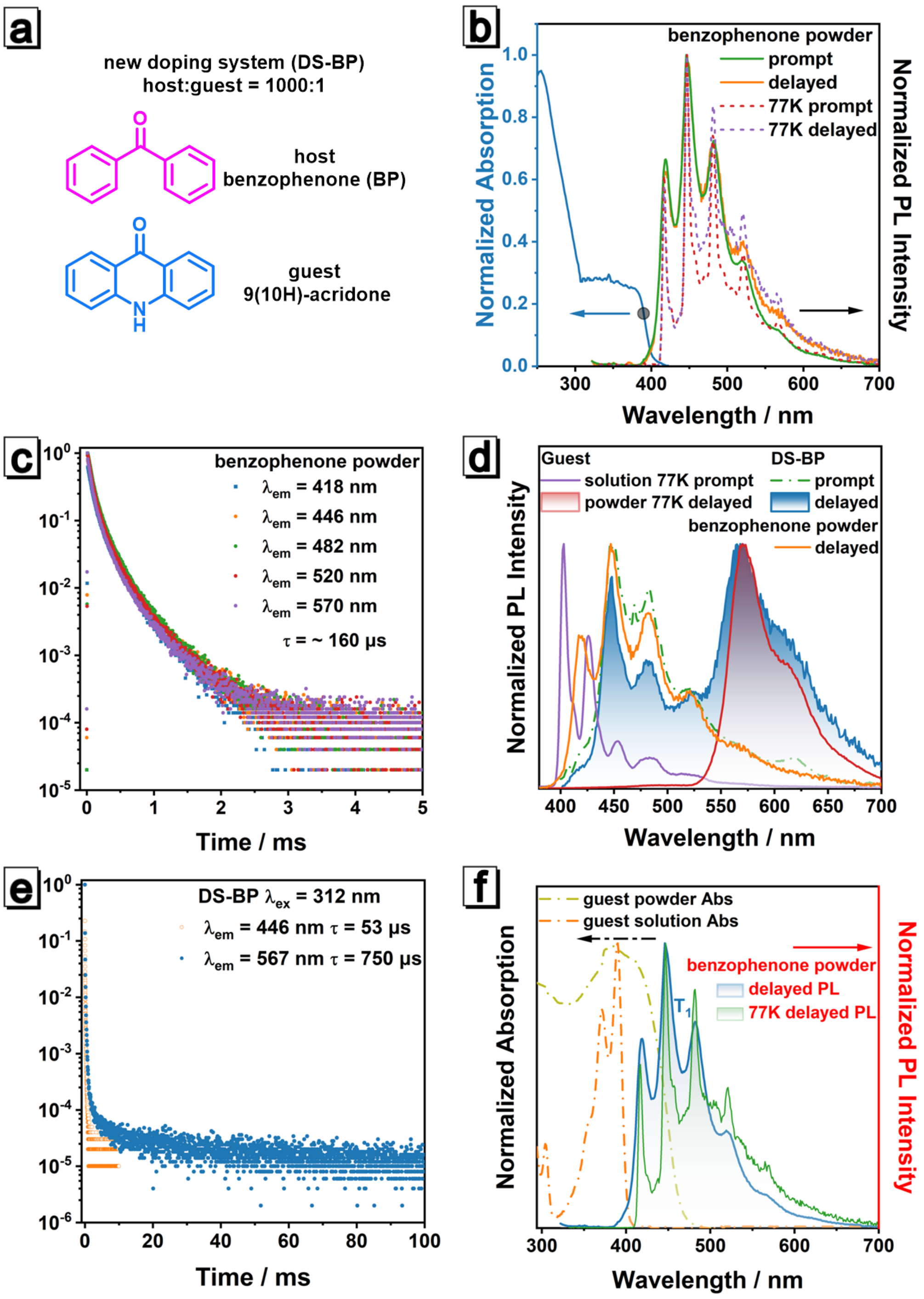


**Supplementary Figure 22** (a) The new doping system **DS-BP** based on benzophenone host. (b) Normalized absorption spectrum, prompt, and delayed PL of benzophenone powder at room temperature and 77 K. (c) Decay curves of delayed emissions of benzophenone powder at room temperature. (d) Comparison of prompt and delayed PL spectra of **DS-BP** with those of **guest** and benzophenone powder. (e) Decay curves of delayed emissions of **DS-BP** at room temperature. (f) Normalized absorption of the powder and the solution of **guest**, and normalized delayed PL spectra of benzophenone powder at room temperature and 77 K.

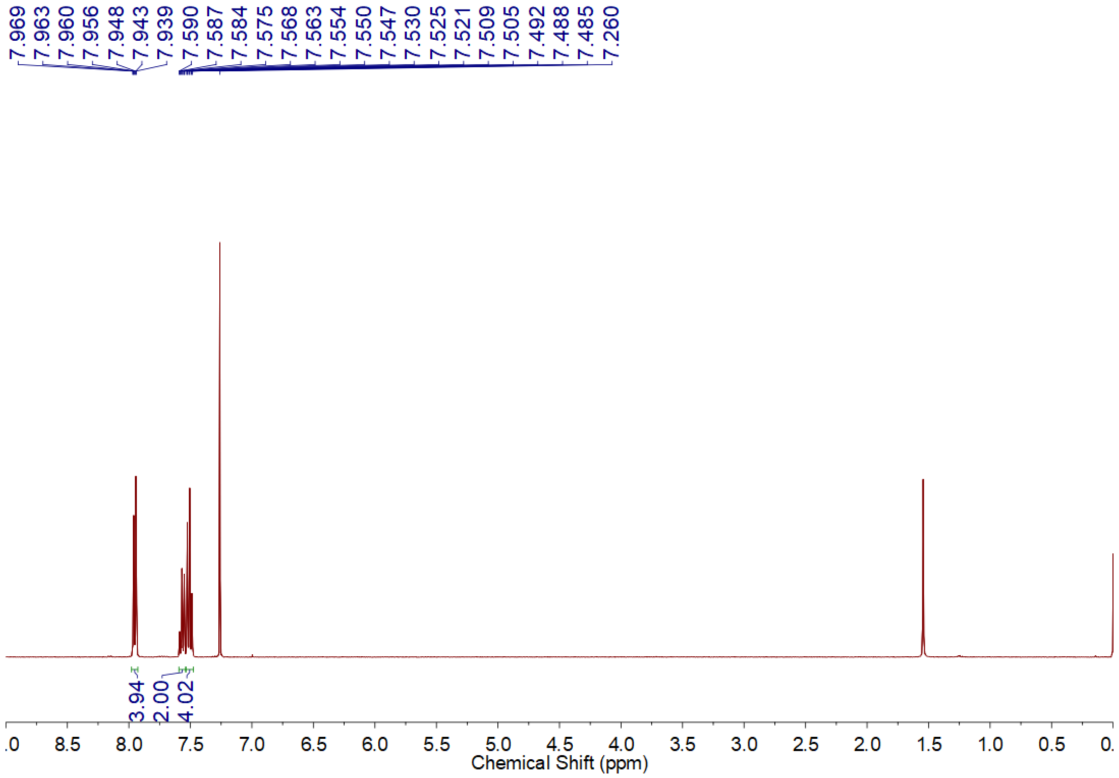


**Supplementary Figure 23** $^{1}H$ NMR of **host**.

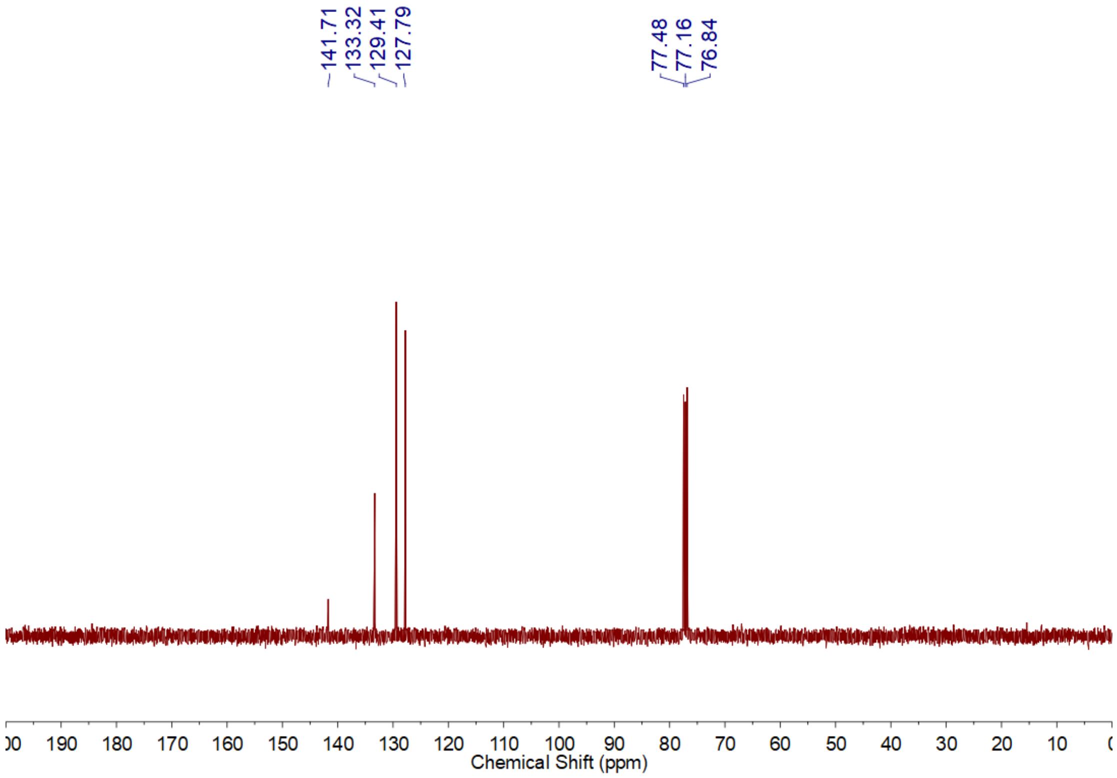


**Supplementary Figure 24** $^{13}$C NMR of **host**.

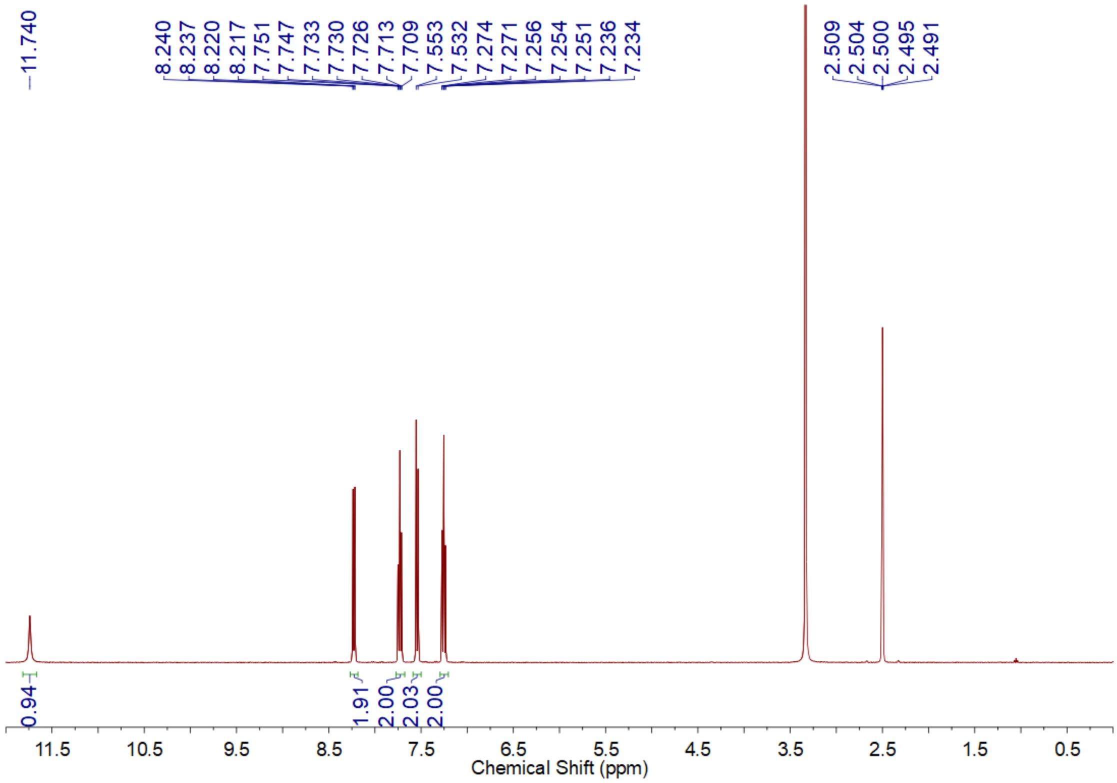


**Supplementary Figure 25** $^{1}$H NMR of **guest**.

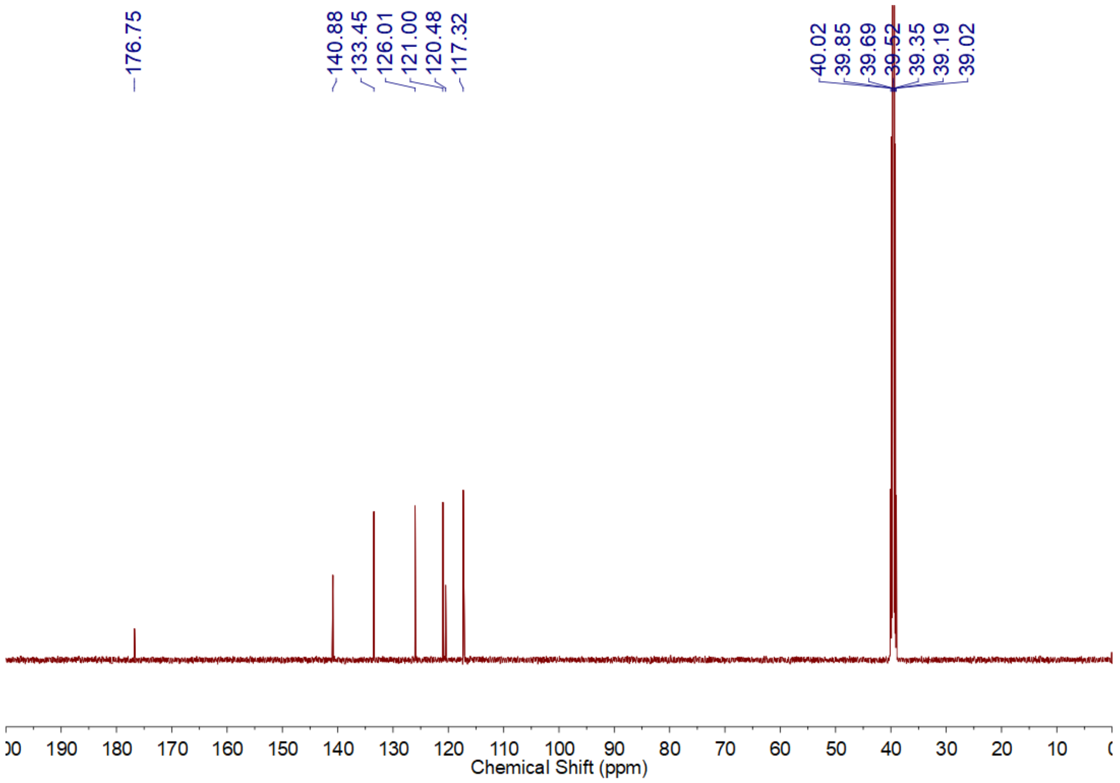


**Supplementary Figure 26** $^{13}$C NMR of **guest**.

**Supplementary Table 1** Comparison of the quantum yields of **host** powder, **guest** powder and different doping systems.

| $\lambda_{ex}$/nm / sample | 374 | 295 | |
|---|---|---|---|
| | afterglow | fluorescence | afterglow |
| **host** | ~0 | 3.93 | 0.48 |
| **guest** | —— | 0.9 | —— |
| **DS-a 1:100** | 1.22 | 2.72 | 2.17 |
| **DS-a 1:1000** | 1.19 | 3.72 | 2.61 |
| **DS-a 1:10000** | 2.66 | 8.98 | 3.27 |
| **DS-g 1:1000** | 0.78 | 12.58 | 2.94 |
| **DS-m 1:1000** | 6.54 | 2.06 | 5.05 |

**Supplementary Table 2** Delayed emission lifetimes in 2-MeTHF solution of **host** (10 μM) at 77 K.

| $\lambda_{em}$/nm | $\tau$/ms | $\tau_1$/ms | proportion of $\tau_1$ | $\tau_2$/ms | proportion of $\tau_2$ |
|---|---|---|---|---|---|
| 361 | 1337.4 | 811.1 | 84.1% | 4128.7 | 15.9% |
| 374 | 1841.2 | 819.0 | 72.7% | 4562.1 | 27.3% |
| 381 | 1786.0 | 817.5 | 74.1% | 4561.2 | 25.9% |

**Supplementary Table 3** Afterglow lifetimes of **DS-a 1:100**, **DS-a 1:1000**, **DS-a 1:10000**, **DS-m 1:1000** and **DS-g 1:1000** under 295/374 nm excitation.

| $\lambda_{ex}$/nm | **295** | **374** | **295** | **374** | **374** | **295** | **374** | **295** | **374** |
|---|---|---|---|---|---|---|---|---|---|
| $\lambda_{em}$/nm | 400 | | 424 | | 452 | 472 | | 480 | |
| **ratio** τ /ms | | | | | | | | | |
| **DS-a 1:100** | 163.4 | 153.8 | 161.9 | 152.2 | 145.0 | 355.8 | 149.3 | 391.7 | 148.6 |
| **DS-a 1:1000** | 136.2 | 134.5 | 132.7 | 133.2 | 133.5 | 353.8 | 138.0 | 379.1 | 135.3 |
| **DS-a 1:10000** | 155.2 | 155.6 | 155.4 | 150.0 | 153.3 | 469.1 | 158.0 | 502.0 | 155.5 |
| | | | | | | | | | |
| **DS-m 1:1000** | 110.9 | 105.6 | 112.1 | 106.0 | –– | 136.9 | 111.5 | –– | –– |
| | | | | | | | | | |
| **DS-g 1:1000** | 144.4 | 130.4 | 152.9 | 132.4 | 137.1 | 333.2 | 141.7 | 342.0 | 150.4 |

## Supplementary References

1. Chen, J., Rahman, N. U., Mao, Z., Zhao, J., Yang, Z., Liu, S., Zhang, Y., Chi, Z., ***J. Mater. Chem. C***, **7**, 8250-8254 (2019).
